# Posterior Reversible Encephalopathy Syndrome in a Bone Marrow Transplant Patient: A Complication of Immunosuppressive Drugs?

**DOI:** 10.14740/wjon932w

**Published:** 2015-08-27

**Authors:** Mohammad A. Hossain, Waqas Jehangir, Qiang Nai, Naureen Jessani, Rafay Khan, Abdalla Yousif, Shuvendu Sen

**Affiliations:** a Department of Internal Medicine, Raritan Bay Medical Center, 530 New Brunswick Ave, Perth Amboy, NJ, USA

**Keywords:** Posterior reversible encephalopathy syndrome, Tacrolimus, Chronic myeloid leukemia, Bone marrow transplantation, Immunosuppressants

## Abstract

Posterior reversible encephalopathy is a complex but well-recognized clinical and radiological entity associated with a variety of benign and malignant conditions including hypertensive encephalopathy, eclampsia, renal failure and immunosuppressive drugs. The pathogenesis is incompletely understood, although it seems to be related to the breakthrough of auto-regulation and endothelial dysfunction. The clinical syndromes typically involve headache, altered mental status, seizures, visual disturbance and other focal neurological signs and radiographically reversible vasogenic subcortical edema without infarction. Here, we report a case of posterior reversible encephalopathy syndrome in a patient with chronic myeloid leukemia who received allogenic bone marrow transplantation (allo-BMT) and immunosuppressive drugs.

## Introduction

Posterior reversible encephalopathy syndrome (PRES) is a well-recognized clinico-neuroradiological transient condition that predominantly affects the cerebral white matter [1]. Early recognition of this condition is of paramount importance because prompt control of blood pressure or withdrawal or correction of underlying condition will cause reversal of the syndrome and prevent irreversible neurological sequels [2]. Here we report a case of PRES in a patient with chronic myeloid leukemia (CML) placed on tacrolimus after receiving bone marrow transplantation.

## Case Report

A 20-year-old male with history of CML with myeloid blast crisis, refractory to chemotherapy, who underwent matched unrelated donor allogeneic stem cell transplant 1 month ago, was admitted in the hospital because of two episodes of generalized tonic clonic seizure at home. Prior to his seizure, he did not have any confusion but he was hypertensive. He was intubated prophylactically in the emergency department because of altered mental status and started on treatment with intravenous lorazepam and levetiracetam. The patient developed accelerated hypertension of 200/106 mm Hg and pressure normalized with calcium channel blocker and intravenous (IV) nitrates. He underwent imaging of the brain with non-contrast computed tomography (CT) which did not demonstrate any acute findings. His post-transplantation course was complicated by viral reactivation (HHV6 and BK) and graft versus host disease (GVHD) involving skin. He was on prednisone and tacrolimus for GVHD. Few days prior to admission, he was diagnosed with adenovirus hemorrhagic cystitis and viremia and started on IV Cidofovir. He was treated with broad spectrum antibiotics, antivirals and antifungals medications. Magnetic resonance imaging (MRI) examination of the brain performed without and with intravenous contrast showed bilateral focal regions of well demarcated subcortical white matter increased signal intensity involving left inferior frontal, temporal and right parietal lobe on the FLAIR T2-weighted examinations with absence of diffusion abnormality consistent with PRES. Tacrolimus level was obtained on the day of seizure and came back highly elevated 71.10 ng/mL (normal reference: 2 - 20 ng/mL). Tacrolimus was discontinued and mycophenolate mofetil was started. No further episode of seizure was observed and the patient became normotensive. He was extubated the day after admission and transferred to cancer center for further management.

## Discussion

PRES is a well-established brain disorder that predominantly affects the cerebral white matter causing focal reversible vasogenic edematous changes involving predominantly the posterior parietal and occipital lobes, and may spread to basal ganglia, brain stem, and cerebellum [1]. Literature reviewed showed that most cases have occurred after hematopoietic or liver transplants.

### Clinical symptoms

This rapidly evolving neurological condition is clinically characterized by headache, nausea and vomiting, seizures, visual disturbances, altered sensorium, and occasionally focal neurological deficit [3-5]. The seizures are usually of generalized tonic-clonic type and may be preceded by visual auras and visual hallucinations, consistent with occipital lobe seizures [5].

### Conditions associated with PRES

PRES has been described in association with severe hypertension, transplantation (allo-BMT, solid organ transplantation), toxemia of pregnancy, autoimmune disease, infection/sepsis/shock, immunosuppressive and cytotoxic drugs and renal failure [6-12] (Table 1).

**Table 1:** Conditions at Risk for PRES [7-12]

Hypertensive encephalopathy
Acute or chronic renal disease
Toxemia of pregnancy
Post-transplantation
Allo-BMT
Solid organ transplantation
Infection/sepsis/shock
Multiorgan dysfunction syndrome
Autoimmune disease
Systemic lupus erythematosus
Wegener’s granulomatosis
Polyarteritis nodosa
Cryoglobulinemia
Immunosuppressive, immunomodulatory, and chemotherapeutic drugs
Bevacizumab
Cisplatin and other platinum based agents
Cyclosporin
Tacrolimus
Cytarabine
Gemcitabine
Interferon-alpha
Intravenous immunoglobulin
Ipilimumab
Methotrexate
Rituximab
Tyrosine kinase inhibitors (pazopanib), sorafenib, sunitinib
Vincristine
Thrombotic thrombocytopenic purpura
Hemolytic and uremic syndrome
Miscellaneous reported associations
Hypomagnesemia [8, 9]
Hypercalcemia [10, 11]
Hypocholesterolemia [12]
Ephedra overdose
Triple-H Therapy
Tumor lysis syndrome
Dialysis/erythropoietin syndrome
Porphyria
Contrast media exposure (cerebral, coronary angiography)

### Pathophysiology

The actual pathogenesis of this clinical syndrome remains unclear, but it appears to be related to disordered cerebral auto-regulation, endothelial dysfunction (endothelial cell swelling, surface marker expression, endothelin release) immune system activation (T cell, related to transplant/microorganism), vascular instability, system/organ hypoperfusion in addition to procoagulant and metabolic effects [1]. Here we mainly discussed the PRES associated with allo-BMT and immunosuppressants. Transplant tolerance is aided by immunosuppression (tacrolimus), limiting acute GVDH and graft rejection mostly after hematopoietic transplantation. PRES incidence is significantly higher after allo-BMT using myeloablative marrow preconditioning and cyclosporine immune suppression, most commonly in the first month of transplantation, remainder during the subsequent year after transplantation. It is the consequence of tumor cell lysis, APC tumor antigen expression, CD4^+^ T cell activation and monocyte/macrophage activation likely develops with subsequent cytokine expression. Increased recognition of PRES with higher doses of chemotherapy could reflect immune response to unique tumor antigen and/or the direct effects of chemotherapy on the endothelial cell. Tacrolimus is a macrolide that reduces peptidyl-prpyl isomerase activity and inhibits calcineurin. It functions by inhibiting T lymphocyte signal transduction and interleukin-2 (IL-2) transcription, thus inhibiting T helper lymphocyte growth and proliferation [13]. It has also a probable direct toxic effect on neurons with axonal swelling and direct capillary endothelial injury causing a blood barrier breakdown may explain the clinical and radiological findings. Seizure associated with PRES may also increase lactate levels and serum CO_2_ with a subsequent increase in vascular permeability which is very much similar to this case [14]. Contributing factors that led to PRES in our patient are the recent allo-BMT, young age, sepsis and higher level of tacrolimus and GVHD. Incidence of PRES with tacrolimus immune suppression appears variable, and association increases with the degree of graft mismatch. Reported rate of tacrolimus associated PRES following allogenic hematopoietic stem cell transplantation was 1.6% [15]. Though it is well established that presence of PRES does not correlate with the blood levels of immunosuppressant, only practical aid for guiding physicians but discontinuation or switch usually results in clinical improvement. In our case the onset of PRES correlates with high serum tacrolimus level but it is also compounded by the other contributing factors including GVHD, recent stem cell transplantation and infection as also discussed by Hammerstorm et al [16].

### Conclusion

Tacrolimus-related PRES is a known but serious complication after bone marrow transplantation where supratherapeutic levels of immunosuppression are required to prevent rejection. A prompt diagnosis based on clinical history and early initiation of treatment are crucial to prevent neurological complications. Withholding or decreasing the dose of tacrolimus in addition to control of hypertension and seizure is mainstay of management. We should be more vigilant and concern regarding this clinical syndrome while patients are on tacrolimus and other clinical conditions associated with PRES.
